# Fabrication of Bacteria Environment Cubes with Dry Lift-Off Fabrication Process for Enhanced Nitrification

**DOI:** 10.1371/journal.pone.0165839

**Published:** 2016-11-03

**Authors:** S. A. P. L. Samarasinghe, Yiru Shao, Po-Jung Huang, Michael Pishko, Kung-Hui Chu, Jun Kameoka

**Affiliations:** 1 Department of Electrical & Computer Engineering, Texas A&M University, College Station, Texas, United States of America; 2 Zachry Department of Civil Engineering, Texas A&M University, College Station, Texas, United States of America; 3 Department of Material Science and Engineering, Texas A&M University, College Station, Texas, United States of America; 4 Department of Biomedical Engineering, Texas A&M University, College Station, Texas, United States of America; MJP Rohilkhand University, INDIA

## Abstract

We have developed a 3D dry lift-off process to localize multiple types of nitrifying bacteria in polyethylene glycol diacrylate (PEGDA) cubes for enhanced nitrification, a two-step biological process that converts ammonium to nitrite and then to nitrate. Ammonia-oxidizing bacteria (AOB) is responsible for converting ammonia into nitrite, and nitrite-oxidizing bacteria (NOB) is responsible for converting nitrite to nitrate. Successful nitrification is often challenging to accomplish, in part because AOB and NOB are slow growers and highly susceptible to many organic and inorganic chemicals in wastewater. Most importantly, the transportation of chemicals among scattered bacteria is extremely inefficient and can be problematic. For example, nitrite, produced from ammonia oxidation, is toxic to AOB and can lead to the failure of nitrification. To address these challenges, we closely localize AOB and NOB in PEGDA cubes as microenvironment modules to promote synergetic interactions. The AOB is first localized in the vicinity of the surface of the PEGDA cubes that enable AOB to efficiently uptake ammonia from a liquid medium and convert it into nitrite. The produced nitrite is then efficiently transported to the NOB localized at the center of the PEGDA particle and converted into non-toxic nitrate. Additionally, the nanoscale PEGDA fibrous structures offer a protective environment for these strains, defending them from sudden toxic chemical shocks and immobilize in cubes. This engineered microenvironment cube significantly enhances nitrification and improves the overall ammonia removal rate per single AOB cell. This approach—encapsulation of multiple strains at close range in cube in order to control their interactions—not only offers a new strategy for enhancing nitrification, but also can be adapted to improve the production of fermentation products and biofuel, because microbial processes require synergetic reactions among multiple species.

## Introduction

Cross-linked hydrogels such as polyethylene glycol (PEG) or collagen are important materials in the biological and medical fields [1–9]. The porous nanostructure of hydrogels is able to transport molecules and provide flexible but stable mechanical characteristics [10]. Recently, these hydrogels have been miniaturized to increase molecular transport in a microscale environment. Many of these microscale hydrogels are fabricated by conventional emulsion and polymerization techniques. However, it is challenging to create the uniform size distribution of hydrogels [11–17]. To address this issue, photolithography [18], imprint lithography [19], and micro-molding [20–21] involving the patterning process for micro-scaling the polyethylene glycol diacrylate (PEGDA) have all been investigated for the roles they play in precise dimension control. The precise patterning of photo-curable hydrogels has significant advantages over conventional emulsification approaches. Multiple layered hydrogel microstructures are also fabricated by flow lithography [22]; however, the complicated control system leads to a low throughput of 3D hydrogel structures. The microfabrication technique for hydrogels also provides a method of encapsulating biological materials such as cells or bacteria. This is of particular interest for its application in medical, biological, and nanotechnology applications. [23–30] There have been many conventional methods reported for encapsulating bacteria in hydrogels [31–32]. Encapsulation of microorganisms within a hydrogel matrix protects the microorganisms from biotic and abiotic stress factors such as contamination, temperature, pH, mechanical, and UV exposure [32–33]. Materials such as poly-L-Lysin [34], gelatin [35], lignin [36], polyuria [37], polyvinyalcohol [37], polyamides [38], and polyacrylates [39] have all been investigated for bacterial encapsulation applications via conventional emulsifications. None of these approaches, however, can precisely encapsulate multiple bacteria within hydrogel structures, precisely. The encapsulation of bacteria via lithography in a microscale PEGDA has previously been demonstrated by researchers [40–41]. Theoretically, low dosage UV does not influence viability of microorganisms [42]. However, when a lithographic approach is used to pattern microstructures three-dimensionally through PEGDA, we discovered that the light-scattering problem rendered it impossible to accurately generate three-dimensional PEGDA structures. The PEGDA square pattern of the first layer can be patterned perfectly without any defects. However, the four small PEGDA squares in the second layer end up being round and connected together in a clover shape, as shown in Figure A in S1 File. This proves that such lithographic approaches cannot be used to encapsulate multiple types of bacteria that are localized precisely and three-dimensionally.

Removing ammonia from wastewater before discharging the treated wastewater back into a natural water body is necessary because excess ammonia stimulates algal growth and impairs the quality of natural waters [43–45]. Nitrification is a two-step process that converts ammonia to nitrate by nitrifying bacteria. In the first step, ammonium (NH_4_^+^) is oxidized into nitrite (NO_2_^-^) by ammonia-oxidizing bacteria (AOB) (see Eq 1). Then, NO_2_^-^ is oxidized into nitrate (NO_3_^-^) by nitrite-oxidizing bacteria (NOB) (see Eq 2) [46].

$$2NH_{4}^{+}+3O_{2}\to 2NO_{2}^{-}+2H_{2}O+4H^{+}$$ (1)

$$2NO_{2}^{-}+O_{2}\to 2NO_{3}^{-}$$ (2)

Successful nitrification relies on synergetic interactions between the AOB and NOB [43–44]. However, the AOB and NOB are slow growers and very sensitive to pH and temperature; additionally, they are easily repressed by many organic and inorganic inhibitors in wastewater [45]. In addition, because of the suspended growth of AOB and NOB in wastewater, the uneven distributions and concentrations of these two cultures commonly lead to inefficient chemical transports between them, making the nitrification process inefficient. Moreover, a toxic intermediate product—nitrite—significantly reduces the performance of the AOB’s ammonia-removal process. To address these issues, we investigated the encapsulation of bacteria in microscale cubes as microenvironment modules where an optimized environment is provided, such that the bacteria could efficiently remove the ammonia from the water. A proximate microenvironment allowed the AOB and NOB to work closely and efficiently, preventing them from being washed away from the treatment system, reducing toxic nitrides, and protecting them from inhibitors; together the result was an enhancement of the ammonia-removal processes.

Our 3D dry lift-off process, with its straightforward and high throughput, is the first system to three-dimensionally localize multiple types of bacteria in PEGDA particles. Compared to the natural environment, 3D bacteria encapsulation can provide a closed and stable living microenvironment that minimizes environmental disturbance. In addition, encapsulating the AOB and NOB separately in particles at specific ratios potentially promotes their synergetic reactions and enhances the nitrification process. To prove this concept, two model strains—AOB and NOB—are used in this study for cell encapsulation.

## Experiment

**Fig 1:**
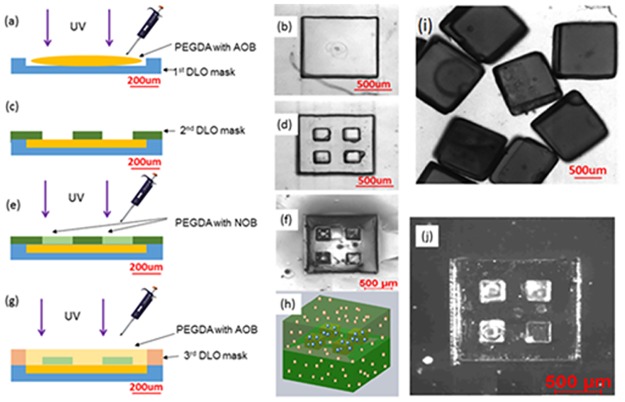
Schematic diagram of the 3D dry lift-off process. (a) A PEGDA solution with AOB was dispensed onto the first DLO mask and cross-liked by 45 seconds of 365nm UV exposure (power). (b) Photo image of the first step. (c) Second PDMS DLO mask was attached to the first PEGDA. (d) Photo image of the second step. (e) PEGDA with NOB medium dispensed onto the second DLO mask and cross-linked. (f) Microscopic image of a micro-cube after the second step. Four square pads were located on the first layer of the PEGDA. (g) Third-layer DLO mask was attached to the second PEGDA layer. The PEGDA solution with AOB was dispensed and cross-linked. (h) Schematic diagram of the PEGDA micro-cube with localized AOB (red dots) and NOB (blue dots). (i) Microscopic image of the PEGDA micro-cube with localized AOB and NOB. (j) Fluorescent microscopic image of a PEGDA micro-cube after the second DLO process. Green fluorescent micro-beads of 1 μm diameter were localized on four small square pads by the DLO process.

PDMS solution is prepared by mixing original PDMS (Sylgard 184 **80**g) solution with **10**g cross-linker solution. PEGDA solution is prepared as follows. PEGDA solution 3 g of PEGDA powder (MW 10000) is dissolved with 10ml of PBS. This solution is mixed with 20μL of photo initiator (30 Wt%). The fabrication process for the PEGDA particles with AOB and NOB is illustrated in Fig 1. The preparation and culture processes for AOB and NOB are detailed in Table A in S1 File. The first and second layers of the PDMS dry lift-off (DLO) mask molds are made by the standard soft lithographic process (shown in Figure B in S1 File). Briefly, an SU8 2075 negative photoresist (MicroChem) was spin-coated onto 3-inch silicon wafers. The lithographic process defined the SU8 2075 negative photoresist as the first-, second-, and third-layer DLO mask molds. A PDMS solution is dispensed on the first- (300 μm thickness), second- (200 μm thickness), and third-layer (300 μm thickness) DLO mask molds. Upon cross-linking, the masks are peeled from the molds. The first-layer DLO mask is placed on the silicon surface, and the PEGDA solution with the AOB bacteria is dispensed and exposed to 365nm UV light (80W) for cross-linking (as shown in Fig 1a). A photographic image of this process is shown in Fig 1b. The second-layer DLO PDMS mask is aligned by marks and placed on the first-layer DLO mask, as shown in Fig 1c. The DLO PDMS mask consists of 120 PEGDA particles in a single wafer. A photographic image of this process is shown in Fig 1d. A PEGDA solution containing NOB bacteria is dispensed onto four small square pads on the surface and cross-linked by UV light exposure, as shown in Fig 1e. The microscopic image of the PEGDA particles after the second-layer patterning is shown in Fig 1f. Clearly, it can be seen that the four small PEGDA squares are patterned precisely on the first layer of the PEGDA. The third DLO mask is aligned and attached to the surface of the second PEGDA layer.

A PEGDA solution with AOB bacteria is then dispensed and cross-linked, as shown in Fig 1g. As the final step, all remaining PDMS masks are removed and the PEG cubes detached from the silicon surface. A schematic diagram of the final 3D PEGDA cube containing the two types of bacteria, localized three-dimensionally, is shown in Fig 1h. These cubes are then submerged in a culture medium. A microscopic image of the collected PEGDA cubes is shown in Fig 1i. Due to the identical refractive indices of the PEGDA for the first, second, and third layers, after the third-layer DLO process, the four small second-layer squares are no longer visible. Thus, the bacteria confinement efficiency in the second layer is investigated by fluorescent micro-beads. The fluorescent image of the PEGDA microstructure with 1 μm fluorescent micro beads localized in four small pads at the second layer of the PEGDA micro-cube is shown in Fig 1j. More than 98% of the fluorescent micro-beads were confined to the four square pads.

## Result and Discussion

**Fig 2:**
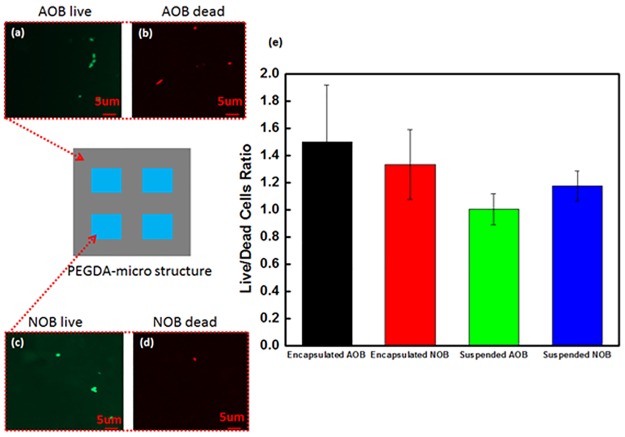
The number of live and dead cells in the micro-cubes. Fluorescent images of (a) the live AOB, (b) the dead AOB, and (c) the live NOB in the PEGDA micro-cubes. The images shown here are in a 50μm x 50μm field of view. (e) Bar graphs of the average ratio of live to dead cells in the PEGDA micro-cubes after UV light exposure and suspended cells in a water solution. There were no obvious differences in the dead/live ratios of the encapsulated versus the suspended cells.

We have investigated the viability of the bacteria by using an Invitrogen LIVE/DEAD^®^ BacLight^™^ [47] because the bacteria is exposed to 365nm UV light for the PEGDA cross-linking with 45 second exposure. Typical fluorescent microscope images of live and dead AOB encapsulated in a PEGDA cube (except for the area containing the center four cubes) after UV light exposure are shown in Fig 2a and 2b, respectively. Fluorescent microscope images of live and dead NOB encapsulated in the four center cubes after UV light exposure are also shown in Fig 2c and 2d, respectively. These images have 50 μm square fields of views. 50 images that include about 500 cells are taken at various locations in each AOB are and NOB area. Then, the number of dead and live cells is counted. The dead-live ratios for these bacteria are averaged for the NOB and AOB in the PEGDA particle and original solution. These ratios are shown as bar graphs in Fig 2e. The standard deviations of cell viability are calculated from these 50 samples of the data obtained from randomly selected locations. The 50% of bacteria is dead in original bacteria solution because the bacteria grow process takes a week and some bacteria is dead during this process. Based on Fig 2e, cells are viable after the UV exposure process; no significant differences are seen in the live/dead ratios calculated with and without UV light exposure. From this information, we can conclude that the influence of the UV light exposure is minimal on the bacteria viability.

**Fig 3:**
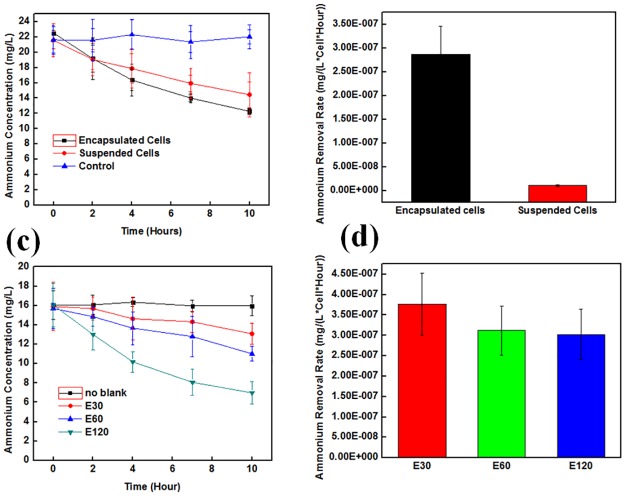
Ammonium removal over time. (a) Comparison of ammonium removal by micro-cubes and suspended samples. The control sample did not contain any bacteria. Ammonium concentration as a function of time. It is obvious from these data that the micro-cube approach had a better nitrate production volume. (b) The ammonium removal rate per single cell for suspended and encapsulated samples. (c) The ammonium removal by different numbers of PEGDA cubes. Ammonium concentration as a function of different numbers of PEGDA cubes. (d) The ammonium removal rate per single cell for encapsulated samples with different numbers of PEGDA cubes.

Ammonium removal by the bacteria in PEGDA particles and liquid suspension are investigated by monitoring the ammonia concentrations (Text A in S1 File). The AOB and NOB PEGDA particles are dispensed in water (Table B in S1 File). Fig 3a shows the ammonium concentrations as a function of time by the suspended bacteria sample and PEGDA particles. From this graph, 120 particles containing 3.0×10^7^ AOB and 1.1×10^7^ NOB cells demonstrated a 25.5% better level of efficiency for removing ammonium than did the suspended samples containing about 6.5×10^8^ AOB and 2.5×10^8^ NOB (Table A in S1 File). The original concentration ratio between AOB and NOB is determined based on the previous report that demonstrated the best result [46]. The single cell ammonium removal rate per hour for the suspended and encapsulated samples is shown in Fig 3b. Based on this bar graph, the efficiency of ammonium removal per single cell (Equation A in S1 File) for the calculation of ammonium removal rate) by the PEGDA particles is about 30 times better than that of the suspended sample. This result is potentially due to the close localization of the NOB and AOB that induced synergetic interaction. Because the NOB were located close enough to the AOB, they could absorb the nitrite made by the AOB and efficiently convert it into nitrate without any negative influences affecting the AOB. The effect of the number of cubes on the efficiency of the ammonia removal is also investigated. Samples of 30, 60, and 120 particles are prepared and used to remove the ammonium. The ammonium concentration curves as a function of time for these samples are shown in Fig 3c. The ammonium reduction rate as a function of time increased by increasing the number of micro-cubes. A sample of ammonium without bacteria is used as a control. The single cell ammonium removal rates per hour for these three samples are shown in Fig 3d. The normalized ammonium removal rates for these samples are almost identical.

**Fig 4:**
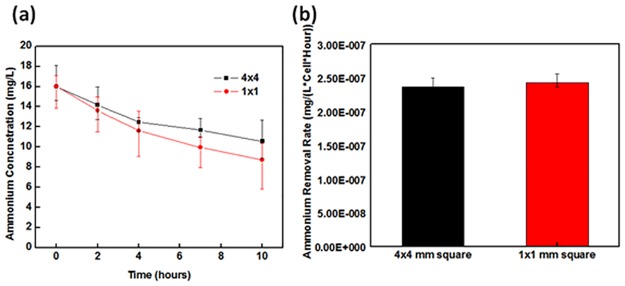
Ammonium removal efficiencies for 1mm x 1mm and 4mm x 4mm PEGDA structures. (a) Ammonium concentrations as a function of time for both samples. (b) Normalized ammonium removal rates for both samples.

**Fig 5:**
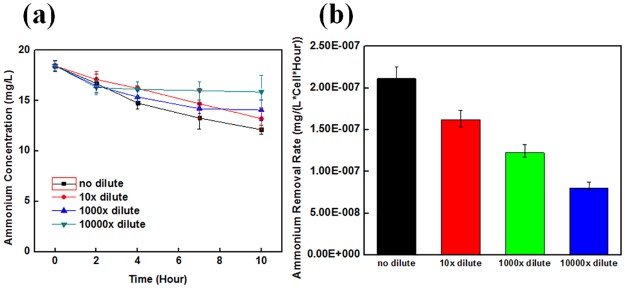
Ammonium removal rate as a function of total cell concentration. (a) Ammonium removal profile as a function of time for different concentrations of bacteria. (b) Ammonium removal rate per cell as a function of bacteria concentration. This graph illustrates the minimum cell concentration required to maximize the efficiency of ammonium removal. Normal solution contains total bacteria of 3.6 x 10^8^.

The size of the PEGDA dimension influences to the ammonia removal efficiency. The ammonia concentration as a function of time for 1mm×1mm and 4mm×4mm square-shaped PEGDA particles is shown in Fig 4a. From this graph, it can be seen that the 1mm×1mm PEGDA particles performed better the 4mm×4mm particles. The normalized ammonium removal rates per single cell for both samples are shown in Fig 4b. The difference is caused by the diffusion effect of ammonium through the PEGDA structures. Larger PEGDA particles require more time for the ammonium to reach the NOB. We have also investigated the dilution effect of bacteria for its effect on ammonia removal efficiency. The ammonium removal profiles as a function of time for no dilution, 1/10, 1/100, and 1/10000 diluted bacteria samples are shown in Fig 5a. The no dilution sample contains total bacteria of 3.6×10^8^. The ammonium removal profiles for the normal, 1/10, 1/1000, and 1/10000 diluted samples clearly are different; a greater level of dilution reduces the total volume of ammonia removed. The ammonium removal rate per hour as a function of the bacteria concentration is shown in Fig 5b. The dilution of bacteria concentration also reduces the normalized ammonium removal performance.

**Fig 6:**
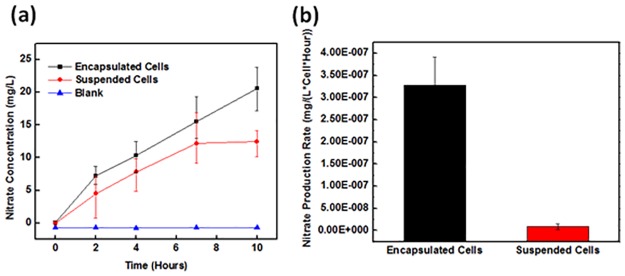
Nitrate production rates by suspended and encapsulated cells. (a) Nitrate production over time by encapsulated cells, suspended cells, and blanks (no cells). (b) Nitrate production rates by encapsulated and suspended cells.

Because nitrite is toxic to AOB, the rapid oxidation of nitrite to nitrate by NOB is critical to stabilizing and improving the efficiency of ammonia removal [43]. Nitrate production levels by suspension and micro-cube sample are shown in Fig 6a. The PEGDA micro-cube and suspended solution samples containing 1.1×10^8^ and 2.5×10^8^ NOB cells, respectively. The normalized ammonia removal rate (expressed as ammonia concentration per cell, per hour) is shown in Fig 6b. As shown in Fig 6b, approximately 30–40% more nitrate is produced by the encapsulated cells. Thus, the nitrate production rate (per cell) of the micro-cube sample is 30 times better than that of the suspended sample.

## Conclusion

We have successfully developed a 3D dry lift-off process to localize AOB and NOB three-dimensionally in PEGDA particles as bacterial microenvironment modules. These encapsulated cells have demonstrated vastly enhanced toxic ammonia removal in water. To the best of our knowledge, this is the first time two types of bacteria have successfully been localized in microscale three-dimensional structures. Cell viabilities are confirmed in the PEGDA particles after UV light exposure during the PEGDA patterning. Our 3D dry lift-off process and design facilitates synergetic bacteria interactions and the rapid diffusion of ammonia from a bulk solution into particles, where it is then converted to nitrite by AOB. The toxic nitride for the AOB becomes readily available in the vicinity of the NOB, and then is localized in four square pads to facilitate further oxidation to nitrate. As a result, the ammonia removal rate per single cell in the PEGDA particles is about 30 times better than in the suspended cell approach. Moreover, in our approach, we can engineer and construct different functions of bacteria at specific location of microparticles through dry lift-off fabrication. Compare to single type encapsulation [48], multiple bacteria encapsulation via dry lift-off lithography can enhance 8-folder high of ammonium digestion rate. This encapsulation approach is applicable to many biochemical reactions involving multiple bacterial strains, such as anaerobic fermentation for bioenergy or valued-product production, and nitrogen removal (nitrification, denitrification, and/or anaerobic ammonia oxidation). Also, this fabrication process is valuable for cancer researches or tissue engineering by immobilizing multiple types of cells in specific locations.

## Supporting Information

S1 File Supporting figures and tables. (DOCX)
