# Supplementary material for: Fabrication of Bacteria Environment Cubes with Dry Lift-Off Fabrication Process for Enhanced Nitrification
Source: PLoS One. 2016 Nov 3;11(11):e0165839. doi: 10.1371/journal.pone.0165839 (PMC5094588; doi:10.1371/journal.pone.0165839)
Supplement: S1 File — (DOCX) [file pone.0165839.s001.docx]

**Supporting Information File S1**

**Figure A: 3D pattering of PEGDA by lithography**

The first layer of PEGDA (1mm square) is patterned by a contact aligner via photo-mask. Then, un-cured PEGDA solution is dispensed on the top of 1^st^ PEGDA layer and four small pads are patterned by lithography as shown in Fig Aa in S1. The side view of this exposure process is shown in Fig Ab in S1. The result of second exposure is shown in Fig Ac in S1. This second exposure process is failed due to the light scattering process. It is obvious that four small squares are not resolved.


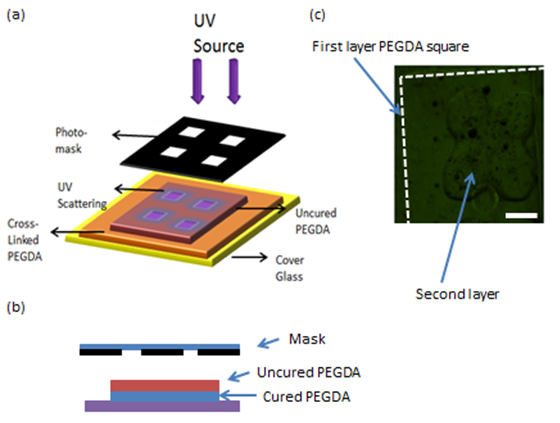


**Fig. A** **3D pattering of PEGDA by lithography.** (a) Schematic diagram of second PEGDA layout exposure process on the first layer PEGDA. (b) Schematic diagram of side view of 2^nd^ PEGDA layer exposure process. (c) Microscopic image of PEGDA with 1^st^ and second layer PEGDA. It is obvious that second layer is not resolved. The scale bar is 500 µm.

**Table A: Bacteria preparation process**

**Strains and growth conditions:**

*Nitrosomonas europaea* is purchased from the American Type Culture Collection (ATCC 19718) and is grown in AOB culture medium and *Nitrobacter winogradskyi* was purchased from the American Type Culture Collection (ATCC 25391) is grown on NOB culture medium. The cells are grown in the dark at 30^o^C at 150 rpm for 3-5 days before harvesting for experimental use. The cells are pelleted by centrifugation (at 10,000g and 4^o^C for 10 minutes) and stored in their respective medium at 4^o^C before using it in the experiment.

**Sample Preparation:**

Water sample used for this experiment includes inorganic carbon source, nitrogen source and no nitrite compounds. The concentrations of other elements are shown in S5. Ammonia and carbon concentrations 20 mg/L and 36 mg/L, respectively, are added into the media during the degradation tests. Encapsulated cells in PEGDA micro-cubes and suspended cells are washed twice with the medium without ammonia ((NH_4_)_2_SO_4_) nor carbon (Na_2_CO_3_). AOB and NOB are incubated separately to reach concentrations of 3.2×108 cells/ml for AOB and 2.0×109 cells /ml for NOB for experimental use. For the cell suspension sample, 2 ml of AOB culture medium solution, 0.15 ml of NOB culture medium solution are mixed with prepared water sample with total volume of 100 ml. About 9.0×108 cells (AOB: 6.5×108 and NOB: 2.5×108) are suspended in 100 ml water sample. For the PEGDA micro-cube sample, 0.8 µl of AOB and 0.05 µl of NOB solutions are used to encapsulate them in PEGDA micro-cubes. The total numbers of cells in 120 PEGDA micro-cubes on a single batch are AOB: 3.0×107, NOB: 1.1×107. Total 4.1×107cells are in 100 ml medium. The numbers of cells in samples are correlated to optical density measurements.

**AOB culture medium recipe**

(NH_4_)_2_SO_4_ (for 50 mM NH^4+^)........................................................4.95 g

KH_2_PO_4_ ..............................................................................................0.62 g

MgSO_4_ . 7H_2_O...................................................................................0.27 g

CaCl_2_ . 2H_2_O.....................................................................................0.04 g

FeSO_4_ (30 mM in 50 mM EDTA at pH 7.0)................................0.5 ml

CuSO_4_ . 5H_2_O....................................................................................0.2 mg

Distilled water...................................................................................1.2 L

Autoclave solution at 121°C for 15 minutes.

**Solution 2:**

KH_2_PO_4_ ..............................................................................................8.2 g

NaH_2_PO_4_ ............................................................................................0.7 g

Distilled water...............................................................................300.0 ml

Bring to pH 8.0 with 10N NaOH. Filter sterilize.

Solution 3 (buffer):

Na_2_CO_3_ anhydrous............................................................................0.6 g

Distilled water.................................................................................12.0 ml

**NOB culture medium recipe**

Solution A (see below).......0.5 ml

Solution B (see below).......0.5 ml

Solution C (see below).......1.0 ml

Solution D (see below).......0.5 ml

Solution E (see below).......0.5 ml

Solution F (see below).......2.0 drops

Distilled water to...........1.0 L

Autoclave at 121°C for 15 minutes.

**Solution A:**

CaCl_2_ ........................2.0 g

Distilled water............100.0 ml

**Solution B:**

MgSO_4_7H_2_O ................20.0 g

Distilled water............100.0 ml

**Solution C:**

EDTA.........................0.14 g

FeSO_4_ . 7H_2_O .................0.5 g

H_2_SO_4_ (conc.) ................0.05 ml

Distilled water............100.0 ml

**Solution D (trace metals):**

Na_2_MoO_4_ . 2H_2_O................0.1 g

MnCl_2_ . 4H_2_O .................0.2 g

CoCl_2_ . 6H_2_O .................0.002 g

ZnSO_4_ . 7H_2_O .................0.1 g

CuSO_4_ . 5H_2_O .................0.02 g

Distilled water..............1.0 L

These ingredients should be dissolved separately and then added together to make 1.0 L.

**Solution E:**

NaNO_2_ .......................41.4 g

Distilled water............100.0 ml

**Solution F:**

K_2_HPO_4_ .......................1.74 g

Distilled water............100.0 ml

**Figure B: Fabrication process for Dry lift off masks**

Three master molds (bottom square, middle small four squares and capping) for 3 dimensional dry lift off (DLO) masks are fabricated by conventional lithographic process. SU8 2075 negative photo resist is spin-coated on top of silicon wafers multiple times followed by a 65^o^C prebake step during each coating process as shown in Fig Ba in S1. Thicknesses of SU8 resist for all mater molds are 500 µm. Silicon wafers with SU8 are exposed to UV via mask aligner for 60 seconds at 14mJ/cm^2^ and post baked at a 65^o^C as shown in Fig Bb in S1. All three master molds baked at 120 ^o^ Celsius for 10 minutes as the final step to harden the structure to be molds as shown in Fig Bc in S1. Dry lift off (DLO) masks are made by dispensing and spin-coating PDMS (Polydimethylsiloxane) to molds and cross-linked by hard baking SU8 2075 for 2 hours as shown in Fig Bd in S1. PDMS DLO masks are peeled from the SU8 2075 master mold for bacteria patterning application as shown in Fig Be in S1.


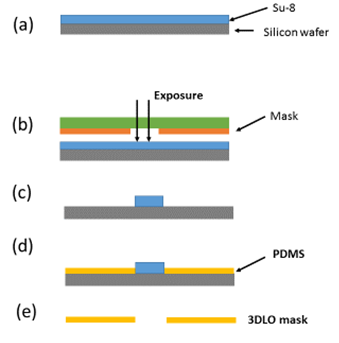


**Fig. B Schematic diagram of the fabrication process for 3D lift off masks made from master molds.** (a) SU8 2075 negative photo resist is spin-coated on silicon wafer. (b) SU8 2075 resist is exposed through a lithographic mask. (c) DLO mask molds after the hard baking. (d) After development process, PDMS solution is spin-coated on the SU8 2075 surface. (e) Cross-linked SU8 2075 is peeled from the patterned wafer for 3D lift off process.

**Text A: Chemical detections**

Liquid samples are collected daily and used for ammonia and nitrate measurements. The concentration of ammonia is measured using phenate method, based on reaction of NH_3_ in alkaline solution with phenate to produce a blue color (indophenol blue). Nitrate (NO_3_^-^) concentration is measured using Dionex 3000 Ion Chromatograph.

**Table B: Detailed chemicals in Water sample**

| **Chemicals** | **Concentration(mg/L)** |
| --- | --- |
| K_2_HPO_4_ | 3.48 |
| MgSO_4_ | 48.8 |
| CaCl_2_ | 10 |
| FeSO_4_ | 2.73 |
| EDTA | 1.4 |
| CuSO_4_ | 6.40E-04 |
| Na_2_MoO_4_ | 0.0428 |
| MnCl_2_ | 7.78E-02 |
| ZnSO_4_ | 2.80E-02 |
| CoCl_2_ | 5.46E-04 |
| Ammonia Concentration | 20 |
| Carbon Concentration | 36 |
| **Encapsulated cells and suspended cells are washed twice by this solution without adding ammonia source ((NH_4_)_2_SO_4_) and carbon (Na_2_CO_3_) before ammonia removal experiment.** | |

**Equation A: The calculation for ammonium removal rate for single cell.**

The single cell ammonium removal rates per hour is calculated by the difference of ammonium concentration in the time period and total number of bacteria encapsulated in hydrogel based on the equation below.

$$Ammonium removal rate for single cell$$

$$=\frac{C_{NH4, fianl-}C_{NH4, initial}}{number of bacteria in one PEG microparticle\times time}$$
